# Indirectly Recognized HLA-C Mismatches and Their Potential Role in Transplant Outcome

**DOI:** 10.3389/fimmu.2014.00210

**Published:** 2014-05-12

**Authors:** Kirsten A. Thus, Liane Te Boome, Jürgen Kuball, Eric Spierings

**Affiliations:** ^1^Laboratory for Translational Immunology, University Medical Center Utrecht, Utrecht, Netherlands; ^2^Department of Hematology, University Medical Center Utrecht, Utrecht, Netherlands

**Keywords:** HLA, transplantation immunology, indirect recognition, epitopes, T-lymphocyte, GvHD, HSCT

## Abstract

HLA-C mismatches are clearly associated to alloreactivity after hematopoietic stem-cell transplantation; in a number of large cohorts, HLA-C mismatches are correlated to an increased risk of acute graft-versus-host disease (GVHD) or even impaired survival. While for HLA-A and -B, both antigenic as well as allelic mismatches are associated with an increased risk of acute GVHD, such an increased risk is only observed for antigenic HLA-C mismatches and not for allelic mismatches. These observations raise the question what sets HLA-C apart from HLA-A and -B. The difference may well be related to the reduced levels of cell-surface expression of HLA-C as compared to HLA-A and -B, possibly due to, among other factors, a limited peptide-binding capacity. This limited peptide-binding capacity may retain HLA-C in the ER and enhance degradation of the HLA-C protein. Once degraded, HLA-C-derived peptides can be presented to the immune system via other HLA alleles and are thus available for indirect recognition. Indeed, such HLA-C-derived peptides have previously been eluted from other HLA alleles. We have recently developed an approach to predict indirect recognition of HLA molecules, by establishing the numbers of predicted indirectly recognizable HLA epitopes (PIRCHES). The number of PIRCHES presented on HLA class I and II (PIRCHE-I and -II, respectively), are highly correlated to clinical measures of alloreactivity, such as acute GVHD. In the present “Hypothesis & Theory,” we reviewed the current knowledge on HLA-C mismatches and alloreactivity. Moreover, we speculate about the role of direct and indirect recognition of HLA-C and the consequences for donor selection in HLA-C mismatched stem-cell transplantation.

## Introduction

HLA-C is a classical HLA class-I protein, thus expressed on nucleated cells and is able to present peptides to T-cells. Like the other classical HLA class-I proteins (HLA-A and -B), HLA-C consists of a polymorphic heavy chain and the non-polymorphic β2-microglobulin. The coding region for the heavy chain is located on chromosome six, in close vicinity of the HLA-B locus. HLA-C and -B alleles are therefore often inherited in non-random combinations, the so-called linkage disequilibrium.

Under normal conditions, HLA-C is expressed at low levels on the cell surface. This low expression level is likely the result of multiple factors: the HLA-C heavy chain messenger RNA is unstable (1); the HLA-C heavy chain does not associate efficiently with the β2-microglobulin (2–4); HLA-C presents a rather restricted repertoire of peptides due to a very restricted α1 domain (5, 6). Due to the restricted peptide repertoire and the inefficient association with β2-microglobulin, HLA-C is often retained within the endoplasmic reticulum (ER) and degraded (4, 6, 7). Next to presenting peptides, HLA-C also serves as a ligand for natural killer (NK) cell receptors: killer immunoglobulin-like receptors (KIR). HLA-C binding to KIRs can act as a negative or positive signal for the NK cells. It is often proposed that the negative signal is the main function of HLA-C and that therefore HLA-C cell-surface expression levels are low (7) [for a comprehensive review regarding the function of HLA-C in relation to KIR, see (8)].

**Figure 1:**
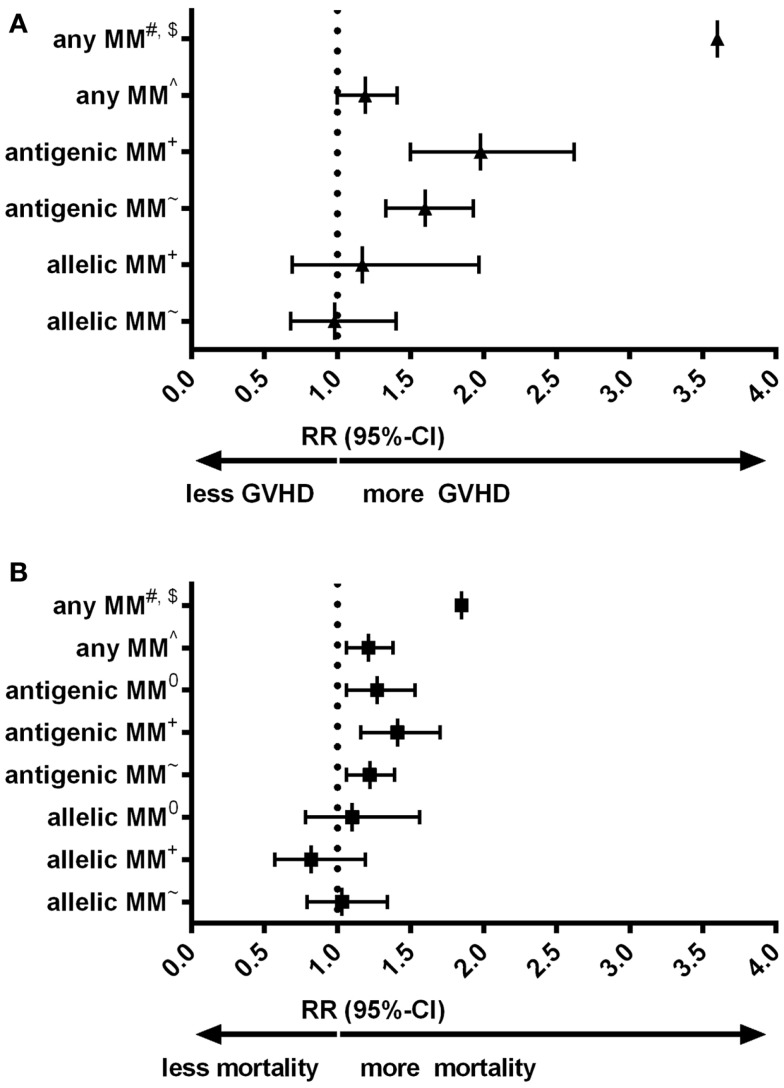
**(A)** Forest plot for relative risk of acute GVHD III-IV of HLA-C mismatches compared to HLA-matched transplants. **(B)** Forest plot for relative risk of mortality of HLA-C mismatches compared to HLA-matched transplants. ^#^: (10) 33 cases of an HLA-C mismatch (either allelic or antigenic, also in combination with other mismatched loci) were compared to 78 10/10 matches. ^$^: Authors indicated hazard ratios. ^∧^: (11) 749 HLA-C mismatches (either allelic or antigenic) were compared to 108 8/8 matches. ^+^: (9) 189 HLA-C antigenic mismatches and 61 HLA-C allelic mismatches were compared to 1243 8/8 matches. ~: (12) 382 HLA-C antigenic mismatches and 96 HLA-C allelic mismatches were compared to 1840 8/8 matches. ^0^: (13) 300 HLA-C antigenic and 57 HLA-C allelic mismatches were compared to 1511 10/10 matches. 10/10 match: donor and recipient matched on high-resolution level for HLA-A, -B, -C, -DRB1, and -DQB1. 8/8 match: donor and recipient matched on high-resolution level for HLA-A, -B, -C, and -DRB1. MM: mismatch. RR: relative risk, relative to either 8/8 or 10/10 matches, as indicated.

Despite the low expression level of HLA-C, HLA-C mismatches are clearly associated to alloreactivity after hematopoietic stem-cell transplantation (HSCT): in a number of large cohorts HLA-C mismatches are correlated to an increased risk of acute graft-versus-host disease (GVHD) or even impaired survival (Figures 1A,B) (9–13). Interestingly, for other HLA class-I mismatches (HLA-A and -B) both low-resolution level (antigenic) as well as high-resolution level (allelic) mismatches are associated with an increased risk of acute GVHD; whereas for HLA-C mismatches this increased risk is only observed for HLA-C antigenic mismatches (9, 12). The effect of HLA-C mismatches on alloreactivity may be explained by NK-cell recognition, however, the exact role of missing KIR ligands in HLA-C mismatched HSCT remains to be elucidated (14). On the other hand, development of acute GVHD clearly involves antigen recognition by T-cells [As reviewed in (15)]. The aim of this “Hypothesis & Theory” paper is to provide a potential explanation for the high immunogenicity of HLA-C antigenic mismatches, despite the low cell-surface expression levels. This potential explanation is based on how T-cell recognition might be involved in the alloreactivity related to HLA-C mismatches.

## T-Cell Alloreactivity

HLA mismatches can lead to T-cell induced alloreactivity via two routes: direct or indirect recognition. Direct recognition is the process where the donor T cell recognizes the intact mismatched HLA molecule on the cell surface of recipient’s cells. Direct recognition is unlikely in the case of HLA-C mismatches because of the low cell-surface expression levels. Indirect recognition occurs when the mismatched HLA protein is processed within the cell and is presented as peptides by HLA molecules. At least 59 peptides derived from HLA-C have been eluted from HLA (16). When the mismatched HLA-C-derived peptides differ from self peptides, they can be recognized by T-cells.

Our group has recently developed an approach to predict indirect recognition of mismatched HLA, by establishing the numbers of predicted indirectly recognizable HLA epitopes (PIRCHES) (17). The number of PIRCHES presented on HLA class I and II, PIRCHE-I and -II respectively, are highly correlated to clinical measures of alloreactivity, such as acute GVHD and transplant-related mortality (Thus et al., manuscripts in preparation).

## HLA-C-Derived PIRCHES

Thus, we hypothesize that the thusfar unexplained substantial alloreactivity of HLA-C mismatches evolves due to indirect recognition of HLA-C. Indirect recognition may furthermore explain the observation that HLA-C antigenic mismatches specifically lead to alloreactivity, as antigenic mismatches likely lead to a higher number of indirectly recognizable epitopes compared to allelic mismatches. To support these hypotheses, we analyzed our local cohort of patients transplanted with an unrelated single HLA-mismatched donor (a 9/10) after non-myeloablative conditioning. All patients and donors were typed for HLA-A, -B, -C, -DRB1, and -DQB1, at ultra-high (4-digit) resolution level, resolving all ambiguities. For retrospective high-resolution HLA-C typing of one HSCT pair, no remaining DNA was available. For this single situation, the high-resolution HLA-typing of this donor–recipient pair was deduced based upon the low-resolution HLA-C typing using the HLA-B/-C association probability (18). The majority of the 48 patients included in these analyses, were transplanted with a mismatch for HLA-C (*N* = 20, 42%, Table 1).

**Table 1:** **The number and percentage of patients, per mismatched locus**.

Mismatch locus	*N* (%)
HLA-A	10 (21)
HLA-B	6 (13)
HLA-C	20 (42)
HLA-DRB1	2 (4)
HLA-DQB1	10 (21)

*We investigated our complete local cohort of patients transplanted with a single HLA-mismatched unrelated donor (a 9/10 match) after non-myeloablative conditioning, for various underlying diseases. The majority (42%) was transplanted with an HLA-C mismatch*.

PIRCHES were determined in the previously described manner (17) with some adaptations; differences in the current study are the incorporation of NetMHCIIPan 2.0, and NetChop for predicting processing of peptides with a processing probability of >0.5 and NetMHCPan 2.4 to select potential binders with an IC50 value <500 nM for predicting PIRCHE-I (19–22).

**Figure 2:**
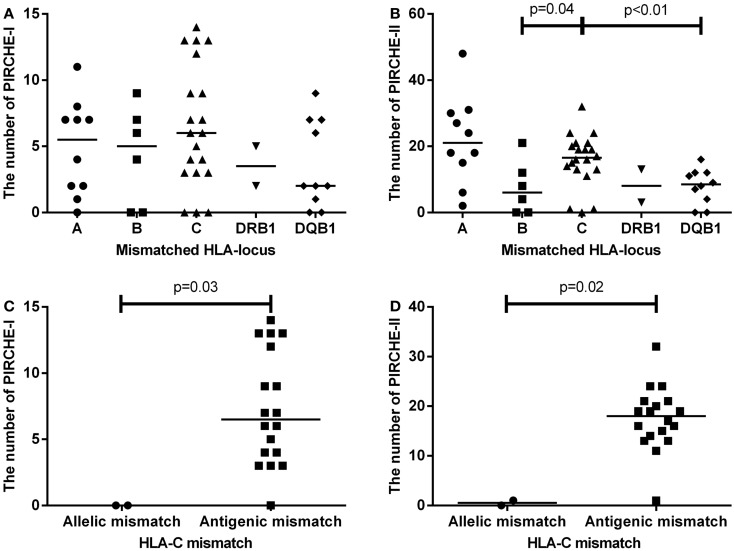
**(A)** The number of PIRCHE-I displayed by the mismatched locus they are derived from. **(B)** The number of PIRCHE-II displayed by the mismatched locus they are derived from. **(C)** The number of PIRCHE-I derived from an HLA-C allelic versus antigenic mismatch. **(D)** The number of PIRCHE-II derived from an HLA-C allelic versus antigenic mismatch. Horizontal lines indicate the median value and the differences between groups were tested with Mann–Whitney *U* tests. Patients with an HLA-C mismatch had a significantly higher number of PIRCHE-II compared to patients with an HLA-B or -DQB1 mismatch. Patients with HLA-C antigenic mismatches, had higher number of PIRCHE-I and -II compared to allelic mismatches.

We first analyzed the numbers of PIRCHE-I and -II separate per mismatched HLA locus (Figures 2A,B). HLA-C mismatches yielded the highest numbers of PIRCHE-I (Figure 2A), although the numbers of PIRCHE-I derived from HLA-C were not significantly different when compared to those derived from the other loci, likely due to the low patient numbers. The number of PIRCHE-II derived from HLA-C were significantly higher than those derived from HLA-B and HLA-DQB1 (Figure 2B, *p* = 0.04 and *p* < 0.01, respectively). The majority of the HLA-C mismatches were antigenic mismatches (*N* = 18, 90%). The abundance of antigenic HLA-C mismatches may explain the high PIRCHE numbers, as the antigenic HLA-C mismatches led to significantly higher numbers of PIRCHES than the allelic mismatches (*p* = 0.03 and *p* = 0.02 for PIRCHE-I and -II, respectively). Allelic HLA-C mismatches always resulted in 0 PIRCHE-I, whereas the number of PIRCHE-II did not exceed 1. Antigenic HLA-C mismatches led to a median of 6 PIRCHE-I (range 0–11), and a median of 18 PIRCHE-II (range 1–32) (Figures 2C,D).

To investigate whether indirect recognition of HLA-C predicts alloreactivity, we selected the HLA-C mismatched transplantations only. We subsequently analyzed whether the risk of alloreactivity is related to the number of PIRCHES instead of the allelic versus antigenic definition. To this end, we redefined the HLA-C mismatches into low or higher number of PIRCHES. We defined 0 PIRCHE-I as low PIRCHE-I, as this was the number of PIRCHES derived from the allelic mismatches, and we defined ≤1 PIRCHE-II as low PIRCHE-II, as 1 was the maximum number of PIRCHE-II derived from the allelic mismatches. Interestingly, we have previously shown that these cut-offs were also the cut off values of the lowest tertiles of HLA-DPB1 derived PIRCHES (manuscript in preparation).

**Figure 3:**
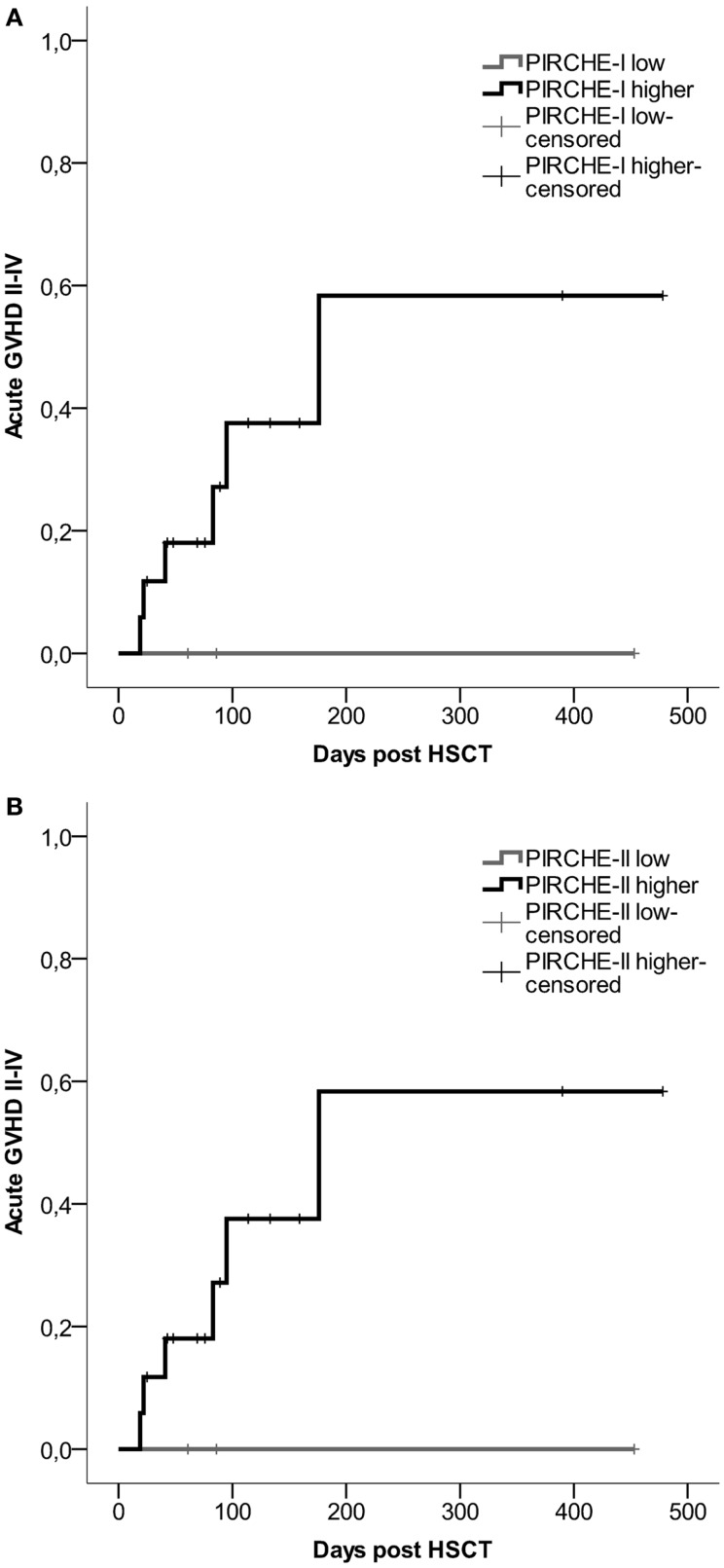
**(A)** Probability of acute GVHD by HLA-C-derived PIRCHE-I low or PIRCHE-I higher. **(B)** Probability of acute GVHD by HLA-C-derived PIRCHE-II low or PIRCHE-II higher. Kaplan–Meier curves were constructed to analyze the probability of developing acute GVHD II-IV for patients in the low (in gray) and higher (in black) PIRCHE groups. Patients with low PIRCHE-I did not develop acute GVHD. Patients with low PIRCHE-II did not develop acute GVHD. Probabilities of acute GVHD II-IV were not significantly different amongst the low or higher PIRCHE groups as tested with log-rank tests. GVHD: graft-versus-host disease; HSCT: hematopoietic stem-cell transplantation.

For all transplant recipients, the numbers of PIRCHES were correlated to acute GVHD development. We observe a trend for patients in the higher PIRCHE-I or -II group having an increased probability of acute GVHD compared to the low PIRCHES (Figures 3A,B). Patients presenting low HLA-C-derived PIRCHE-I or -II (*N* = 3) did not develop acute GVHD. This difference is, although striking, not significant, likely due to low patient and event numbers (six events). The number of PIRCHES is not associated to the severity of acute GVHD in this cohort, although such an association requires a larger study population; we observed only three cases of clinically severe acute GVHD (grade III-IV).

## Potential Implications for Donor Selection

For HSCT donor-selection procedures, potential donors are at first mainly typed on a low to intermediate resolution level for HLA-A, -B, and -DRB1. Based on donor–recipient matching for these loci, a limited number of donors are selected for further high-resolution typing, which includes typing of the other loci. For patients with rare HLA-B/-C associations, it will be very challenging to find a donor matched for both HLA-B and -C, due to the strong linkage disequilibrium between HLA-B and -C. As HLA-B matching is considered earlier in the donor-selection procedure than HLA-C, patients with rare HLA-B/-C associations are more frequently transplanted with an HLA-C mismatch. These HLA-C mismatches are often antigenic mismatches, as can be expected from the observed HLA-B/-C associations (18). Our data indicate that these antigenic HLA-C mismatches very frequently lead to high numbers of PIRCHES, due to the low level of homology between the two mismatched alleles. We propose that a mismatch for HLA-B may be considered in these cases, as in some situations the HLA-B mismatches can lead to lower numbers of PIRCHES than HLA-C mismatches.

**Figure 4:**
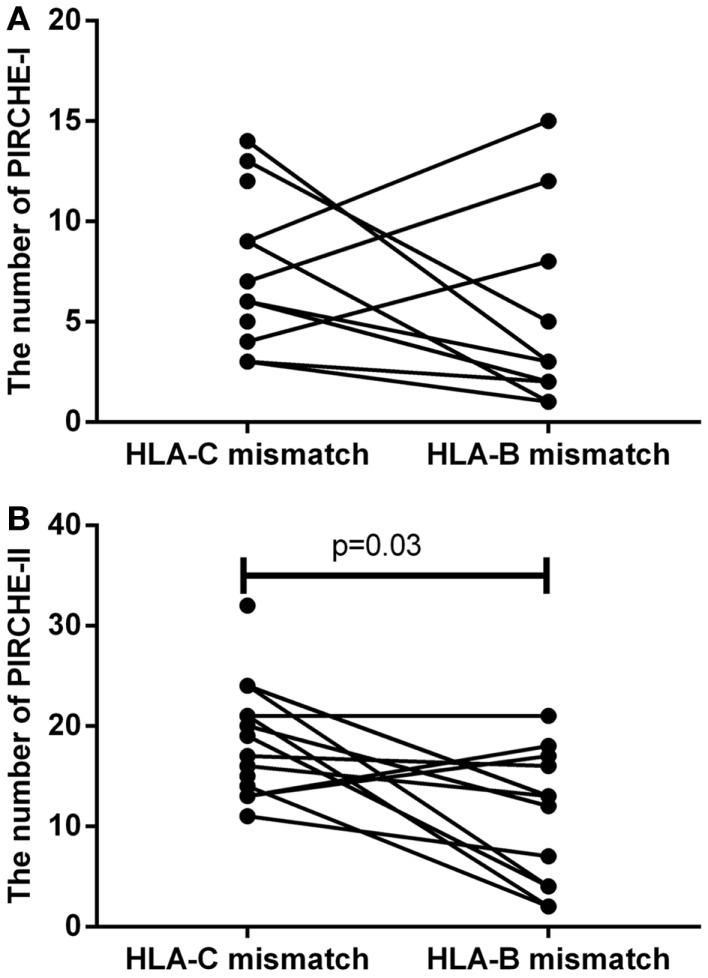
**(A)** The number of PIRCHE-I for the selected HLA-C mismatch, compared to a potential HLA-B mismatch. **(B)** The number of PIRCHE-II for the selected HLA-C mismatch, compared to a potential HLA-B mismatch. For 17 patients with high numbers of mismatched HLA-C-derived PIRCHE-I and -II, we analyzed whether we could potentially find a mismatched HLA-B alternative donor. We found that in 7 (54%) of these cases, we could reduce the number of PIRCHE-I with this strategy and in 9 (69%) of these cases we could reduce the number of PIRCHE-II. The numbers of PIRCHE-II are significantly lower when we would have chosen the potential HLA-B mismatch instead of the selected HLA-C mismatch (Wilcoxon matched-pairs signed ranked test,*p* = 0.03).

To support the above-mentioned option, we performed a theoretical analysis. To this end, we analyzed the possibility to identify an alternative 9/10 mismatched donor for those HLA-C mismatched cases that had an increased probability of acute GVHD (i.e., PIRCHE-I > 0 and PIRCHE-II > 1, *N* = 17), using haplotype frequency tables (18). For these patients, we aimed at a theoretical HLA-B mismatch instead of an HLA-C mismatch. For 13 (76%) patients, we could identify a potential HLA-B mismatched donor (Table 2). In 7 (54%) of these cases, the HLA-B mismatch led to a lower number of PIRCHE-I than the selected HLA-C mismatched donor, and in 9 (69%) of the cases the HLA-B mismatch yielded a lower number of PIRCHE-II than the HLA-C mismatch (Figures 4A,B). The numbers of PIRCHE-II related to an HLA-B mismatch instead of an HLA-C mismatch, are significantly reduced for the HLA-B mismatched cases (*p* = 0.03). Thus, HLA-B mismatches can lead to a lower probability of indirect recognition than HLA-C mismatches. We hypothesize that the effect of HLA mismatches does not depend on a locus-specific effect, but is rather related to the resulting PIRCHES.

**Table 2:** **HLA-typing of the patient, selected HLA-C mismatch, and potential HLA-B mismatch alternative**.

Patient ID	Patient HLA-typing										Selected HLA-C mismatch			Potential HLA-B mismatch		
	HLA-A		HLA-B		HLA-C		HLA-DRB1		HLA-DQB1		HLA-C donor	PIRCHE-I	PIRCHE-II	HLA-B donor	PIRCHE-I	PIRCHE-II
1	01:01	02:01	15:01	51:01	03:03	**15:02**	13:01	15:01	06:02	06:03	07:02	13	16	NA		
2	02:01	–	40:01	51:01	**03:03**	03:04	09:01	13:02	03:03	06:04	05:01	0	1	NI		
4	01:01	03:01	08:01	*47:01*	07:01	**03:04**	03:01	14:01	02:01	05:03	06:02	4	13	40:01	8	18
10	24:02	24:02	15:01	*44:03*	03:03	**05:01**	11:01	12:01	03:01	03:01	04:01	3	24	44:02	2	4
11	02:01	11:01	07:02	18:03	**02:02**	07:01	11:04	15:01	03:01	06:02	07:02	13	32	NA		
14	03:01	32:01	07:02	15:01	07:02	**03:03**	07:01	13:01	02:02	06:03	01:02	4	15	NA		
16	02:01	29:02	*44:04*	51:01	16:01	**15:02**	10:01	11:01	05:01	03:01	14:02	14	19	44:03	3	4
23	01:01	02:01	08:01	15:01	**03:03**	07:01	03:01	04:01	02:01	03:01	03:04	0	1	NI		
25	03:01	11:01	07:02	*18:01*	07:02	**12:03**	11:01	13:01	03:01	06:03	07:01	9	21	38:01	15	21
29	02:01	–	35:01	*51:01*	**01:02**	04:01	13:02	15:01	06:02	06:04	15:02	5	11	27:05	0	7
30	02:01	03:01	*13:02*	35:01	**02:02**	04:01	04:01	07:01	02:02	03:02	06:02	6	16	27:05	3	13
31	02:01	11:01	15:01	*51:01*	04:01	**12:03**	01:01	04:01	03:02	05:01	03:04	7	13	39:01	12	17
33	02:01	24:02	*40:01*	57:01	06:02	**07:02**	07:01	13:02	03:03	06:04	03:04	9	17	07:02	1	16
34	02:01	–	15:01	44:02	**03:04**	05:01	04:01	04:04	03:01	03:02	03:03	0	0	NI		
36	02:01	03:01	07:02	*07:02*	07:02	**02:02**	04:04	15:01	03:02	06:02	07:02	7	19	27:05	0	0
40	01:01	02:01	*15:01*	38:01	**04:01**	12:03	13:01	13:02	06:03	06:04	12:03	12	14	35:03	0	2
44	11:01	24:02	35:01	*35:03*	04:01	**03:03**	04:07	12:01	03:01	–	04:01	3	21	15:01	1	2
46	11:01	68:01	07:02	*27:05*	**02:02**	07:02	01:01	07:01	03:03	05:01	01:02	6	20	44:02	2	12
47	01:01	02:01	*18:01*	27:05	02:02	**05:01**	11:01	15:01	03:01	06:02	07:01	13	24	44:02	5	13
48	01:01	68:01	44:02	51:01	07:04	**14:02**	01:01	04:04	03:02	05:01	15:02	3	19	NA		

*For all our HLA-C mismatched cases, we analyzed the numbers of PIRCHE-I and -II. We next investigated whether we could in theory (based on known haplotypes) identify an HLA-B mismatch instead of an HLA-C mismatch. In this table, HLA-typing of the patient is displayed, in bold: the actual HLA-C mismatched allele and in italic: the potential HLA-B mismatched allele. The HLA-typing of the (potential) donors is only displayed for the mismatch, as the other alleles have the same typing. NA: potential HLA-B mismatched alternative not available; NI: potential HLA-B mismatch not investigated, as the number of HLA-C-derived PIRCHES was low*.

## Discussion

HLA-C mismatches lead to substantial alloreactivity, despite the low cell-surface expression levels of HLA-C. Particularly, antigenic HLA-C mismatches lead to high risks of complications (Figure 1). In our local cohort of patients transplanted with a single HLA mismatch, we show that HLA-C mismatches lead to higher numbers of indirectly recognizable epitopes (PIRCHES) than when mismatches are located on other loci (Figure 2). Furthermore, patients presenting HLA-C-derived PIRCHE-I or more than one HLA-C-derived PIRCHE-II are at a higher risk of developing acute GVHD. Indirect recognition of HLA-C mismatches may therefore provide an explanation for the alloreactive complications observed after HLA-C mismatched transplants.

In theory, HLA-C allelic mismatches may lead to direct recognition by donor T-cells, as the T-cell receptor (TCR) contact residues likely remain similar among allelic mismatches. The polymorphisms in allelic mismatches will mostly reside within the peptide-binding groove, and can thus lead to different peptide presentation repertoires. Because of the self-HLA restriction of the TCR, the T cell may still bind to the allelic mismatch and can then recognize the different peptide repertoire as foreign. With antigenic mismatches, the self and allogeneic HLA will contain large numbers of polymorphic residues, and the TCR may not bind to the mismatched allogeneic HLA anymore. When the TCR cannot bind to the allogeneic HLA, recognition of the mismatch will not occur. Therefore, direct recognition seems more likely in the case of allelic mismatches. In line with this suggestion, previous *in vitro* studies have proposed that one should rather mismatch largely (antigenic) instead of only for a small number of polymorphic residues (allelic) (23). The latter study clearly showed that the chance of the donor developing cytotoxic T-lymphocytic precursors *in vitro* was more likely in less polymorphic mismatches, suggesting a greater probability of direct recognition by donor T-cells *in vivo*. However, as HLA-C cell-surface expression is low, development of direct recognition is less probable. In line with this assumption, the previously reported HistoCheck model for direct recognition of HLA class I mismatches, did not predict alloreactive complications after HLA-C mismatched HSCT (nor for HLA-A and -B mismatches) (24). Similarly, the scores obtained with this direct recognition model showed no correlation to acute GVHD development in our cohort (*p* = 0.97). These observations further support the hypothesis that indirect recognition may be an important route of HLA-C mismatches evoking alloreactivity.

HLA-C cell-surface expression is low due to, among other factors, a limited peptide presentation profile and subsequent unstable association with β2-microglobulin. This instability leads to prolonged HLA-C presence in the ER and finally degradation of the protein. When HLA-C is degraded, it can thereafter be presented on other HLA proteins as peptides. Indeed, HLA-C-derived epitopes are frequently diluted from other HLA alleles (16). These HLA-C-derived PIRCHES can lead to alloreactivity. As HLA-C allelic mismatches lead to a low number of PIRCHES and antigenic mismatches to a high number of PIRCHES, indirect recognition of mismatched HLA-C may explain the risk related to antigenic HLA-C mismatches and the absence of this relationship for allelic HLA-C mismatches.

Recently, another study explained the absence of immunogenicity of HLA-C allelic mismatches by the predominance of the HLA-C*03:03/03:04 mismatch combination in this group (25). In this study, a negative impact on clinical outcomes was observed for HLA-C antigenic mismatches and any mismatch on HLA-A, -B, or -DRB1, whereas HLA-C*03:03/03:04 mismatches had similar outcomes as HLA-A, -B, -C, and -DRB1 matched (8/8) transplantations. In contrast, HLA-C allelic mismatches other than HLA-C*03:03/03:04 did lead to an increased probability of acute GVHD. These data may also be explained by the indirect recognition model, as the HLA-C*03:03/03:04 mismatch leads to a difference in only one amino acid, and therefore likely yields a low number of indirectly recognizable epitopes. Indeed, in our cohort, two patients were transplanted with an HLA-C*03:03/03:04 mismatch, leading to low PIRCHE-I and -II (Table 2).

HLA-C mismatches can not only lead to T-cell recognition; B-cell recognition may alternatively lead to alloreactivity upon HLA-mismatched HSCT. The development of HLA-C specific antibodies is correlated to complications after HLA-mismatched organ transplantation (26). HLAMatchmaker is a well-validated *in silico* tool for analyzing such HLA-specific antibody responses (27, 28). The number of eplets defined by HLAMatchmaker is correlated to the antibody reactivity against mismatched HLA (29). Although HLA antibodies may play a role in HSCT outcome, the HLAMatchmaker algorithm is not suitable to predict GVH-directed alloreactivity in HLA-mismatched HSCT (30). Particularly for HLA-C, a correlation between GVHD and HLAMatchmaker scores may be unlikely; low cell-surface expression of HLA-C also limits the potential of binding by HLA-C specific antibodies to the mismatched alleles. Indeed, in our small cohort of HLA-C mismatches, HLAMatchmaker scores are also not correlated to acute GVHD development (*p* = 0.77).

HLA-C mismatched donors are more frequently selected than HLA-B mismatched donors, due to the previously mentioned donor-selection procedures. We have proposed that HLA-B mismatches may in some situations lead to a lower probability of indirect recognition than HLA-C mismatches, and that therefore an HLA-B mismatch may be preferred. Although some studies indicate a particularly strong effect of HLA-B mismatched transplantations on detrimental outcomes (13); literature remains inconclusive regarding a higher risk of HLA-B mismatches compared to other mismatched loci (9, 11, 12). Before implementing the proposed strategy of selecting donors with the lowest numbers of PIRCHES, regardless of the locus that is mismatched, the effect of PIRCHES per mismatched locus should be studied in a large cohort. Such studies would also allow investigations on the risk of alloreactivity due to direct recognition in cases with zero PIRCHES, reflecting the absence of indirect recognition.

To summarize, in this “Hypothesis & Theory” paper, we investigated whether indirect recognition of HLA-C mismatches may explain the risk of alloreactivity in the context of the relatively low cell-surface expression level of HLA-C. We observed a high number of HLA-C-derived PIRCHES in the case of antigenic HLA-C mismatches. These high numbers of PIRCHES seem to be correlated to an increased acute GVHD risk. We next investigated whether selection of an HLA-B mismatched donor might lead to lower numbers of indirectly recognizable epitopes compared to the selected HLA-C mismatch. Indeed, for a number of patients, we could identify a potential lower immunogenic alternative. It might thus be preferable to select a mismatch that leads to the lowest number of PIRCHES, instead of avoiding mismatches on a specific locus, although this requires confirmation. This strategy may reduce the risk of alloreactive complications. We further propose that future studies investigating the effect of HLA-C, and other mismatches, on alloreactivity after HSCT with different stem-cell sources, need to be conducted in large cohorts in order to verify the clinical relevance of our hypothesis.

## Conflict of Interest Statement

The University Medical Center Utrecht has filed a patent application on the prediction of an alloimmune response against mismatched HLA.
